# Adaptive host responses to infection can resemble parasitic manipulation

**DOI:** 10.1002/ece3.10318

**Published:** 2023-07-15

**Authors:** Camilla Håkonsrud Jensen, Jacqueline Weidner, Jarl Giske, Christian Jørgensen, Sigrunn Eliassen, Adèle Mennerat

**Affiliations:** ^1^ Department of Biological Sciences University of Bergen Bergen Norway

**Keywords:** gigantism, hormone strategy, host compensation, host–parasite coevolution, parasite manipulation

## Abstract

Using a dynamic optimisation model for juvenile fish in stochastic food environments, we investigate optimal hormonal regulation, energy allocation and foraging behaviour of a growing host infected by a parasite that only incurs an energetic cost. We find it optimal for the infected host to have higher levels of orexin, growth and thyroid hormones, resulting in higher activity levels, increased foraging and faster growth. This growth strategy thus displays several of the fingerprints often associated with parasite manipulation: higher levels of metabolic hormones, faster growth, higher allocation to reserves (i.e. parasite‐induced gigantism), higher risk‐taking and eventually higher predation rate. However, there is no route for manipulation in our model, so these changes reflect adaptive host compensatory responses. Interestingly, several of these changes also increase the fitness of the parasite. Our results call for caution when interpreting observations of gigantism or risky host behaviours as parasite manipulation without further testing.

## Graphical abstract

When studying the behaviour and physiology of infected hosts, it can be challenging to disentangle adaptive host responses from parasitic manipulation. Here, we build a model explicitly accounting for hormonal responses in juvenile hosts and linking them to foraging behaviour in the presence of a predator. Via optimisation modelling we show that higher host growth and increased risk‐taking during foraging emerge even in the absence of manipulation, thus representing a pre‐existing pathway within the host that parasites may be selected to exploit.

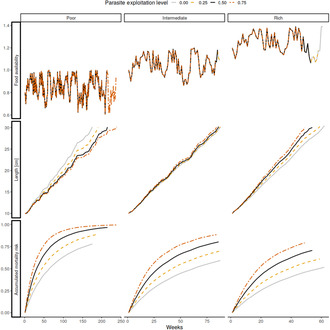

## 1 INTRODUCTION

Hosts and parasites interact antagonistically with each other and many of their traits result from a co‐evolutionary arms race (Brunner et al., 2017; Hudson et al., 2006). In hosts, traits for the avoidance of, and resistance against, parasites (see Table 1 for glossary) are under selection, as evidenced by the wide repertoire of adaptive pre‐ and post‐infection defences. These include reducing infection risk by, for example avoiding certain areas and types of foods (Hutchings et al., 2001), disgust or fear of parasites (Oaten et al., 2009; Prokop et al., 2010) or prophylactic offspring care (Mennerat et al., 2009). Other behaviours occur post‐infection, like grooming, behavioural fever and self‐medication (de Roode et al., 2013; Lefèvre et al., 2009). Hosts can also partly compensate for the detrimental effects of infection via increased foraging effort involving greater risk‐taking (Klein, 2003; Milinski, 1990; see also Hite et al., 2020). In addition to behavioural defences, organisms have an immune system that protects against and fights infections. Immune defences are costly and often traded off against other necessary functions such as growth and reproduction (Poulin et al., 1994; Sheldon & Verhulst, 1996). Hosts may also respond to parasitism by shifting their life histories in adaptive ways, for example by reproducing earlier in the presence of parasites that strongly compromise future reproduction (Ebert et al., 2004; Gabagambi et al., 2020; Minchella & Loverde, 1981). Finally, if neither resistance nor tolerance of the parasite is possible, host suicide may be adaptive if it increases inclusive fitness (Humphreys & Ruxton, 2019; Poulin, 1992); infected eusocial insects have for example been observed to move away from their relatives to die in solitude (Heinze & Walter, 2010).

**TABLE 1:** Glossary.

Changes following infection	Changes in host phenotype (behaviour, physiology, morphology) following a parasitic infection
Manipulation	Phenotypic changes in the host induced by parasitic infection that are adaptive for the parasite, but maladaptive for the host
Compensation	Adaptive phenotypic changes in the host that compensate for some of the detrimental fitness effects of infection
(Host) Resistance	Avoiding or clearing infection
(Host) Tolerance	The ability of the infected host to limit the fitness impact of infection
(Parasite) Exploitation level	The proportion of the host's energy drained by the parasite, relative to the host's standard metabolic rate (see Equation 1)
Virulence	The reduction in host fitness that is due to parasitic infection

Certain parasites, referred to as manipulative parasites, induce changes in host phenotype that increases their own fitness while being counter‐adaptive for the host (Holmes & Bethel, 1972; Poulin, 1995; Thomas et al., 2005). Host manipulation has been the focus of hundreds of studies and is now recognised as a widespread adaptive strategy for parasites (Poulin & Maure, 2015) and one of the best examples of extended phenotype (Dawkins, 1982). The changes in host phenotype following infection range from altered host behaviour or morphology resulting in increased predation rates (e.g. *Schistocephalus solidus* infecting copepodites; Hafer & Milinski, 2016; altered behaviour in roach *Rutilus rutilus* infected with *Ligula intestinalis*; Loot et al., 2001; see also Barber et al., 2000; changes in eye stalk colouration and shape of snails infected with *Leucochloridium* spp.; Wesołowska & Wesołowski, 2014), to gigantism with increased host growth and/or reserves (e.g. *Daphnia magna* infected by *Pasteuria ramosa*; Ebert et al., 2004). These modifications can also be accompanied by physiological changes in hormone levels or in the central nervous system of the host (Escobedo et al., 2005; Klein, 2003).

When host physiology and behaviour change following infection, however, it can sometimes be difficult to assess whether the change is adaptive for the parasite, the host, or is a ‘by‐product’ of the infection. The issue fostered decades of research aimed at testing the adaptive consequences of host manipulation for hosts and for parasites (Poulin, 2021). Caution is warranted, as appearances can be misleading and only experimental work can allow to disentangle cause from consequence (Poulin & Maure, 2015). Besides, most studies of host manipulation have focused on its adaptive value, whereas the underlying proximate mechanisms have largely been overlooked. Identifying the manipulation factors of parasites has been repeatedly called for (Herbison et al., 2018; Poulin & Maure, 2015); hormones, neurotransmitters or symbionts are among the proposed candidates (Herbison, 2017). For example, infection by the parasitic acanthocephalan *Polymorphus paradoxus* in the gammarid *Gammarus lacustris* leads to increased serotonin levels and associated changes in host phototaxis (Maynard et al., 1996; Perrot‐Minnot et al., 2014). But in most other cases of suspected or established host manipulation, there is still a need to determine which pre‐existing pathways, within the host, parasites might be exploiting (Helluy, 2013; Helluy & Thomas, 2010; Lefèvre et al., 2009). The aim of this paper was to explore whether parasites could be selected for exploiting the hormonal responses of hosts to infection. There are several ways in which host responses to the energetic cost of infection could have fitness consequences for parasites. First, whenever predation risk for the host decreases with size, hormone‐mediated enhancement of host growth (through the growth hormone function) could reduce mortality risk for the host and therefore also its parasites. Second, upregulating the appetite of infected hosts (through the orexin function) would make the host forage more actively and be more exposed to predators. This would have opposite consequences on parasite fitness, depending on whether it is trophically or directly transmitted. Finally, increasing the metabolic rate of the host (through the thyroid hormone function) might affect mortality in opposite ways, either by increasing maximum oxygen uptake and thus improving the efficiency of predator escape movements or by increasing the metabolic rate of the host, which would in turn require higher foraging activity and higher risk exposure.

In this study, we incorporate current knowledge of the physiological regulation of feeding and juvenile growth of fish in a model, to test (1) whether some of the host phenotypic changes often attributed to parasite manipulation (e.g. higher growth rates, higher risk‐taking) can arise as adaptive plasticity in the host, as a compensatory response to the energetic costs of parasitism, (2) how optimal host responses to these costs vary according to environmental quality, and (3) whether these changes in the host could also benefit parasites. Using optimisation modelling, we start by testing whether the energetic costs of parasitism alone can lead to hormone‐mediated increases in host growth, body condition and exposure to predation. To do so, we compare the optimal responses of fish hosts experiencing differing levels of parasite exploitation. By simulating three levels of food availability, we then test how the optimal host responses to parasite exploitation differ across environments. Finally, we explore how parasite exploitation level relates to fitness, either for a parasite still developing in its host or for a trophically transmitted parasite ready to leave its intermediate host.

## 2 MATERIALS AND METHODS

We use an optimisation model of hormonal regulation of growth in fish (Jensen, Weidner, Giske, et al., 2020; Jensen, Weidner, Jørgensen, & Eliassen, 2020; Weidner et al., 2020) to study how host growth and behaviour respond to the energetic costs of parasite infection. The model captures the flow of energy through the fish, from foraging and digestion to metabolic activities and growth, while the endocrine system regulates host energetics and mediates trade‐offs with survival. The fish in our model should be seen as juvenile, as for the sake of simplicity we do not consider reproduction or reproductive investment. For each timestep, the model uses stochastic dynamic programming (Clark & Mangel, 2000; Houston & McNamara, 1999) to maximise host survival until adulthood. It does so by finding the optimal combination of hormone levels given two internal and one external state of the fish: stored reserves [J], body length [cm] and food availability [dimensionless].

Here we give an overview of the main features of our model. A complete description of all parameters, variables and equations is also available in Supporting Information. Our approach differs from Dynamic Energy Budget (DEB) models in the sense that it explores adaptive changes in growth rates under varying circumstances. For a longer discussion of our approach compared to DEB models, please see Weidner et al. (2020).

### 2.1 Endocrinology

The endocrine system of the fish regulating feeding and growth is here represented by three simplified main functions: the growth hormone function (GHF), orexin function (OXF) and thyroid hormone function (THF). GHF affects growth rate, OXF appetite, while THF regulates both standard metabolic rate (SMR) and maximum oxygen uptake (Weidner et al., 2020). In the model, fish hormone levels change between distinct timesteps up to a given maximum. The effect of the GHF in a timestep is defined by the proportion of the current GHF level (*γ*) to the maximum GHF level (*γ*_max_). Proportions are also used for THF and OXF and found in the model as (*τ*/*τ*_max_) and (*α*/*α*_max_), respectively.

### 2.2 Metabolism

A standard metabolic rate (SMR, *P*_SMR_) depending on the total weight of the fish (structural weight and reserves) and regulated by the THF is calculated. To translate the THF level (relative to the maximum level) into an effect of THF on SMR, we include an additional factor (*k*_THF_SMR_):

(1) $$P_{\mathrm{SMR}}=\left[1+\left(\frac{\tau }{\tau_{\max}}-0.5\right)\cdot k_{\mathrm{THF}\_\mathrm{SMR}}\right]\cdot P_{\text{standard}}$$

Here, τ [ng mL^−1^] is the current THF level, $\tau_{\max}$ [ng mL^−1^] is the maximum THF level, $P_{\text{standard}}$ [J min^−1^] is the standard metabolic rate based on total weight ($W=W_{\text{structure}}+W_{\text{reserves}}$ [g]) at $\tau_{\max}/2$ and $k_{\mathrm{THF}\_\mathrm{SMR}}$ [dimensionless] is the effect that THF has on $P_{\text{standard}}$. Calculations of SMR are based on Clarke and Johnston (1999).

The energy from metabolism can either be allocated to growth or stored in reserves. The amount of energy stored in reserves depends on the amount of stored energy when the fish enters the timestep (*R*(t)). Increases in reserve size are due to energy from foraging (*I*). Energy allocated to growth (*C*_growth_), foraging (*P*_foraging_) and metabolic costs decrease the size of reserves. These metabolic costs include the SMR (*P*_SMR_), energetic costs of digesting food (SDA, *P*_SDA_) and conversion costs linked to converting metabolites from food to storage molecules in reserves (*P*_reserves_) or from reserves to building blocks for structural growth (*P*_growth_). To scale the size of reserves to the timesteps used by the model, energy expenses must be multiplied by the length of a timestep (*t*_duration_):

(2) $$\begin{array}{c} R\left(t+1\right)=R\left(t\right)-C_{\text{growth}}\\ +\left(I-P_{\mathrm{SDA}}-P_{\mathrm{SMR}}-P_{\text{foraging}}-P_{\text{parasite}}-P_{\text{growth}}-P_{\text{reserves}}\right)\cdot t_{\text{duration}} \end{array}$$

Here $R\left(t\right)$ and $R\left(t+1\right)$ are the reserves R [J] at the beginning and end of the timestep t. Bioenergetic rates must be multiplied by the duration of a timestep, $t_{\text{duration}}$ [min]. The expression $\left(I-P_{\mathrm{SDA}}-P_{\mathrm{SMR}}-P_{\text{foraging}}\right)$ can be viewed as the energetic surplus available [J week^−1^] after accounting for metabolism, digestion and foraging activity.

One main assumption in the model is that survival and physiology are linked via respiration. This approach is built on Priede (1985) as well as empirical studies of the trade‐offs between energy acquisition rates and swimming performance in growing Atlantic silversides (*Menidia menidia*, Billerbeck et al., 2001; Lankford et al., 2001). Oxygen uptake is also influenced by THF. Similarly to metabolic calculations, oxygen uptake (*A*_max_) is based on a standard oxygen uptake rate (*A*_standard_). At THF levels of $\tau_{\max}/2$, oxygen uptake is identical to standard uptake. Up‐ and downregulation are controlled by adaptive THF levels in the distinct timesteps. A factor translating THF proportions to the actual effect of THF on oxygen uptake is included (*k*_THF_scope_):

(3) $$A_{\max}=\left[1+\left(\frac{\tau }{\tau_{\max}}-0.5\right)\cdot k_{\mathrm{THF}\_\text{scope}}\right]\cdot A_{\text{standard}}$$

Here, $A_{\text{standard}}$ [J min^−1^] is the maximum O_2_ uptake at $\tau_{\max}/2$ and $k_{\mathrm{THF}\_\text{scope}}$ [dimensionless] is the effect THF has on $A_{\text{standard}}$. During our simulations, $k_{\mathrm{THF}\_\mathrm{SMR}}$ is slightly higher than $k_{\mathrm{THF}\_\text{scope}}$ (see Table S1). Calculations of maximum oxygen uptake are based on Claireaux et al. (2000).

In the model, we compare the total oxygen use (*P*) from all aerobic metabolic processes with the maximum oxygen uptake, following Holt and Jørgensen (2014). Oxygen use combines the effects of SMR (*P*_SMR_), foraging (*P*_foraging_), SDA (*P*_SDA_) and cost of conversion to reserves (*P*_reserves_) and growth (*P*_growth_). The more oxygen the fish uses relative to maximum oxygen uptake, the less is available for escape, and the more vulnerable the fish will be to predation.

### 2.3 Foraging behaviour

Foraging is initiated by the OXF. In the absence of OXF, the fish will not take up food or use energy for swimming to search for food. Any increase in OXF induces swimming and food intake (*I*). In the model, we assume that foraging activities result in food uptake, thus in environments with low food availability more energy is allocated to swimming. Searching for food without finding is not included. The regulation of foraging behaviour by OXF resembles the control of appetite by the ‘hunger hormone’ ghrelin (Dimaraki & Jaffe, 2006) and the neuropeptide orexin. In the model food intake depends on the SMR of structural tissue (*P*_structural_), the proportion of OXF (*α*/*α*_max_) and a coefficient translating the effect of OXF on intake (*k*_OXF_):

(4) $$I=\frac{\alpha }{\alpha_{\max}}\cdot k_{\mathrm{OXF}}\cdot P_{\text{structure}}$$

Here, α [pg mL^−1^] is the current OXF level, $\alpha_{\max}$ [pg mL^−1^] is the maximum possible OXF level, $k_{\mathrm{OXF}}$ [dimensionless] is the effect OXF has on intake and $P_{\text{structure}}$ [J min^−1^] is the SMR at $\tau_{\max}/2$ based on the structural weight of the fish.

Given a certain appetite, the desired food intake (foraging behaviour, *B*_foraging_) is calculated. The model environment is defined by a certain food availability for the fish. There will always be some food, but when food availability is low the fish must spend more time foraging to reach the same target intake (I [J min^−1^]):

(5) $$B_{\text{foraging}}=\frac{I}{P_{\text{stucture}}\cdot E}$$

Here, $B_{\text{foraging}}$ [dimensionless] is the foraging activity required to reach I for a given $P_{\text{structure}}$ [J min^−1^] and a food availability *E* [dimensionless].

The energetic cost of foraging (*P*_foraging_) is the product of the foraging behaviour, SMR based on total weight (*P*_standard_) and a scaling constant (*k*_foraging_):

(6) $$P_{\text{foraging}}=k_{\text{foraging}}\cdot B_{\text{foraging}}\cdot P_{\text{standard}}$$

### 2.4 Growth

Our model host is seen as a growing, juvenile fish. Growth, controlled by the GHF, is the allocation of energy to new structural tissue (∆*W*_structure_). This requires for metabolites from ingested food to be converted into building blocks for new somatic tissue. GHF is expressed as a proportion of the maximum level of GHF (*γ*/*γ*_max_) and multiplied by a constant (*k*_growth_). Fulton's condition factor (Lambert & Dutil, 1997) is used to convert fish length to structural weight.

(7) $$\Delta W_{\text{structure}}=\left(\frac{\gamma }{\gamma_{\max}}\right)\cdot k_{\text{growth}}\cdot W_{\text{structure}}$$

Here, γ [ng mL^−1^] is current GHF level, $\gamma_{\max}$ [ng mL^−1^] is maximum possible GHF level, $k_{\text{growth}}$ [week^−1^] is the maximum limit for proportional increase in structural body mass in one timestep [weeks], $W_{\text{structure}}$ [g] is structural weight calculated from length using Fulton's condition factor (Lambert & Dutil, 1997). Thus, a higher γ leads to a higher growth per timestep.

The product of the increase in structural weight (∆*W*_structure_) and the energetic value of body structure (Anthony et al., 2000; Fernandez et al., 2009; Holdway & Beamish, 1984) is used as a proxy of energetic cost of growth (*C*_growth_). This energetic cost is drawn from food intake. In timesteps when food intake is too low, the absence of GHF is equivalent to no investment into structural tissue and reserves are drained.

### 2.5 Environment

Environments tend to vary gradually, which is often reflected in the fact that current food availability is correlated with that in the near past and future. We incorporate these aspects in our model by adding temporal autocorrelation to food availability (Ripa & Lundberg, 1996). The fish hosts respond to these fluctuations by adjusting their feeding behaviour, growth rate and metabolism. When the conditions permit it, the fish may build energy reserves that they can draw from in times of scarcity (Jensen, Weidner, Giske, et al., 2020). Different food availabilities are assumed to be normally distributed with environments. Environments with total lack of food are excluded. In poor environments food is harder to find, thus fish spend more time searching and use more energy on foraging. In these environments, mortality risk is higher as fish can be detected by predators while searching for food. Fish have to adapt to current food availabilities as moving to other environments is not part of the model.

### 2.6 Host mortality

Our model fish hosts experience different types of mortality, combined into one total instantaneous mortality rate (M [year^−1^]):

(8) $$M=m_{\text{fixed}}+M_{\text{size}}+M_{\text{foraging}}+M_{\text{scope}}+M_{\text{foraging}\times \text{scope}}$$

These five mortality components are affected differently by hormone function levels and fish body length: (1) size‐independent mortality ($m_{\text{fixed}}$ [year^−1^]), (2) size‐dependent mortality ($M_{\text{size}}$ [year^−1^]), (3) foraging‐related mortality ($M_{\text{foraging}}$ [year^−1^]), (4) scope‐related mortality ($M_{\text{scope}}$ [year^−1^]) and (5) active‐while‐vulnerable mortality component ($M_{\text{foraging}\times \text{scope}}$ [year^−1^]). The size‐independent mortality $m_{\text{fixed}}$ is unaffected by fish length or hormone function levels and is kept at a stable, low level. This low level is chosen as we assume that most of the mortality affecting a small fish is highly dependent on size. Size‐dependent mortality $M_{\text{size}}$ decreases with increasing fish length (see Equation S13). Foraging mortality $M_{\text{foraging}}$ is connected to the foraging activity of the fish ($B_{\text{foraging}}$), which is affected by the food availability of the environment the fish is currently in as well as the OXF level of the fish. For example, if we have two individuals with the same OXF levels and one experiencing low and the other high food availability, then the individual with poor food availability will also experience higher foraging mortality (see Equation S14). The scope‐related mortality $M_{\text{scope}}$ is affected by the ratio between the used oxygen (P) and the maximum oxygen uptake set by THF ($A_{\max}$). It is important to note that THF does not only increase aerobic scope but also the actual O_2_ use through the positive effect of THF on SMR. In other words, a high $M_{\text{scope}}$ means that the fish has a lower potential for escaping a predator (see Equation S15). Finally, the active‐while‐vulnerable mortality component $M_{\text{foraging}\times \text{scope}}$ represents the interaction between foraging and scope mortality. It can be viewed as the fish's potential to escape a predator while foraging, where a higher interaction mortality equates to a poorer potential for escape (see Equation S16).

Hormone levels affect host survival in the following ways (Weidner et al., 2020): First, predation risk for fish generally decreases with size, hence more GHF triggering faster growth reduces mortality risk in the long run. Second, fish with higher OXF levels are more actively foraging and thus more exposed to predators. Finally, THF affects mortality in opposite ways by: (1) increasing maximum oxygen uptake, which makes it easier to escape predators, and (2) by increasing metabolic rate, which requires more oxygen and energy and thus higher foraging activity and risk exposure.

In addition to mortality due to predation, the model incorporates a negative effect of starvation on host survival. Here host survival *S* [week^−1^] follows a negative exponential that depends on total mortality *M* [year^−1^], as well as on relative energy reserves (*R*/*R*_max_) and a coefficient of starvation *k*_starvation_ [dimensionless]. If *R* drops below *k*_*s*tarvation_·*R*_max_ fish survival rapidly declines with relative energy reserves (*R*/*R*_max_):

(9) $$S=e^{-M/52}\cdot \left(\frac{1}{k_{\text{starvation}}}\right)\cdot \left(\frac{R}{R_{\max}}\right)$$

The optimisation process is based on state‐dependent programming and stochastic dynamic optimization (Clark & Mangel, 2000; Houston & McNamara, 1999) to find optimal hormone concentrations yielding the highest survival for the model, growing fish host (for details, see Supporting Information).

#### 2.6.1 Parasite exploitation of host

In our model, we make no assumptions about the life history of the parasite, or whether it is a micro‐ or macroparasite. Within‐host competition is also not explicitly modelled as we make no assumption regarding the number or diversity of parasites infecting the host. For ease of reading, we will here use parasite in the singular form.

The only characteristic of the model parasite is that it takes energy from the host at a certain rate (described below). There is no explicit effect of parasitism on host life history, behaviour or survival, except that the increased energetic demands due to infection may have knock‐on consequences for host mortality, physiology or behaviour.

The rate at which energy is diverted by the parasite [J min^−1^] is set to be proportional to the metabolic rate of the host:

(10) $$P_{\text{parasite}}=P_{\text{structure}}\cdot k_{\text{parasite}}$$

where the coefficient *k*_parasite_ [dimensionless] is the exploitation level of the parasite (ranging between 0 for uninfected hosts to 0.75 for heavily infected hosts) and *P*_structure_ [J min^−1^] is the structural metabolic rate of the fish. Following Weidner et al. (2020) this structural metabolic rate is the product of body mass by an oxygen consumption rate [J min^−1^ g^−1^] under an intermediate level of THF (*τ*_max_/2 [ng mL^−1^] where *τ*_max_ is the maximum THF level [ng mL^−1^]). One of the aims of this study is to compare host responses for different exploitation levels. For the sake of simplicity here, these exploitation levels *k*_parasite_ are kept constant throughout each separate simulation.

#### 2.6.2 Host response to parasites

The model fish has no means of getting rid of the parasite; its only option is to adjust the hormonal regulation of growth and behaviour, ultimately affecting juvenile survival.

The fish host may cover the energetic cost of being parasitised by increasing food intake *I* [J min^−1^] or draining energy from reserves *R* [J]. The host's reserves at the next timestep (*t* + 1) depend on foraging behaviour and energy allocation in the current timestep:

(11) $$\begin{array}{c} R\left(t+1\right)=R\left(t\right)–C_{\text{growth}}\\ +\left(I–P_{\mathrm{SDA}}–P_{\mathrm{SMR}}–P_{\text{foraging}}–P_{\text{parasite}}–P_{\text{growth}}–P_{\text{reserves}}\right)\cdot t_{\text{duration}} \end{array}$$

where *C*_growth_ is the energy incorporated into new structural tissue [J], *I* is intake, *P*_SDA_ is the energetic cost of digesting food [J min^−1^], *P*_SMR_ is the standard metabolic rate under influence of THF [J min^−1^], *P*_foraging_ is the foraging cost [J min^−1^], and *P*_growth_ and *P*_reserves_ are the energetic conversion costs from intake to growth and from reserves to growth [J min^−1^], respectively. Bioenergetic rates are multiplied by the duration of a timestep, *t*_duration_ [min]. Further details can be found in Weidner et al. (2020) where we explore the energetic costs of growth, including conversion costs, in more detail. The only difference between the model presented here and the one used in Weidner et al. (2020), Jensen, Weidner, Giske, et al. (2020) and Jensen, Weidner, Jørgensen, & Eliassen (2020) is the addition of the term *P*_parasite_ representing the rate at which energy is diverted from the host by the parasite (Equation 11).

#### 2.6.3 Experimental simulations

To investigate whether the nature or direction of optimal host responses to parasitism depend on habitat quality, we simulated three groups of individual fish experiencing three different levels of food availability: (1) poor food availability resembling a poor natural environment, (2) intermediate food availability and (3) rich food availability, where conditions arguably reflect ad libitum feeding, for example in the laboratory. Prior to experimental simulation, all individual fish were first optimised to the same wide environmental range of food availabilities spanning all three levels described above.

## 3 RESULTS

The optimal response in fish hosts infected with a parasite diverting energy was to shift hormone levels, which resulted in changes spanning from altered growth rates to modified foraging behaviour and thus exposure to predation.

### 3.1 Physiological and behavioural changes in the fish host

**FIGURE 1:**
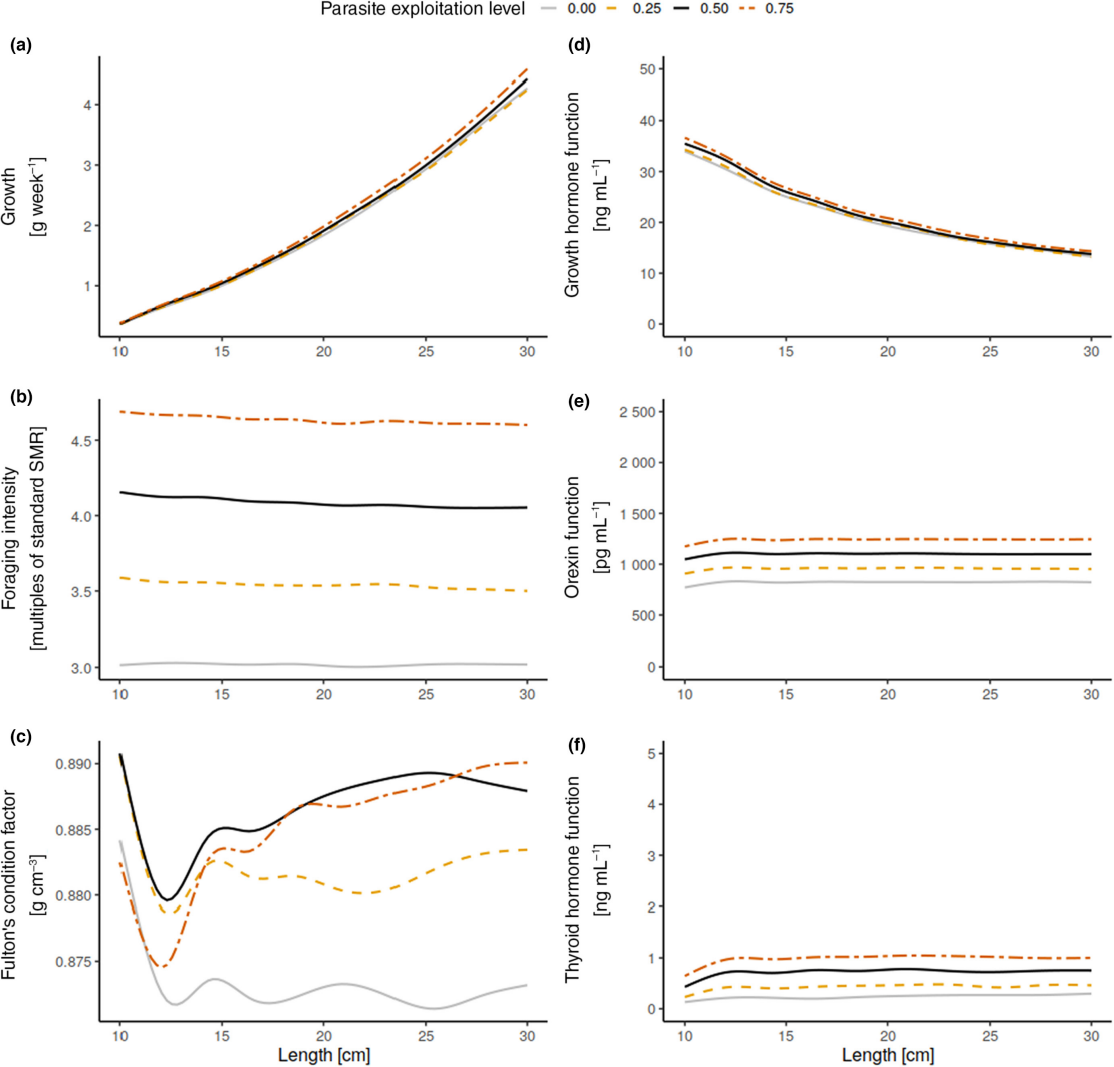
(a) Mean host growth, (b) foraging intensity and (c) Fulton's condition factor [100·(total weight/length^3^)] for different parasite exploitation levels. These emerge from optimising (d) growth hormone function (GHF), (e) orexin function (OXF) and (f) thyroid hormone function (THF) levels in our model for each of the four exploitation levels (see Section 2 for details). Lines are smoothed using a generalised additive model for ease of reading.

Fish harbouring parasites with a higher exploitation level experienced higher energetic costs and compensated with increased foraging intensity (Figure 1b). This was a result of elevated appetite, caused by upregulation of the orexin function (OXF; Figure 1e). Higher parasite exploitation level also increased optimal levels of the thyroid hormone function (THF; Figure 1f), which in turn led to higher metabolism and increased maximum oxygen uptake (following Equations 1 and 3).

Higher foraging intensity and metabolism are expected given the additional energy demand from hosting a parasite. More surprisingly, growth hormone function (GHF) levels and consequently host growth increased with parasite exploitation level (Figure 1a,d, but only in relatively rich environments, see below). Infected hosts also stored more energy in their reserves: At the beginning of the juvenile growth period, the mean Fulton's condition factor [100·(total weight/length^3^)] was variable, but as fish hosts grew it stabilised at higher levels for fish that had parasites with higher exploitation level (Figure 1c). Higher condition, foraging activity, metabolism and growth, however, come at the cost of an increased predation risk (Figure 3a).

### 3.2 Optimal host strategies under different levels of food availability

**FIGURE 2:**
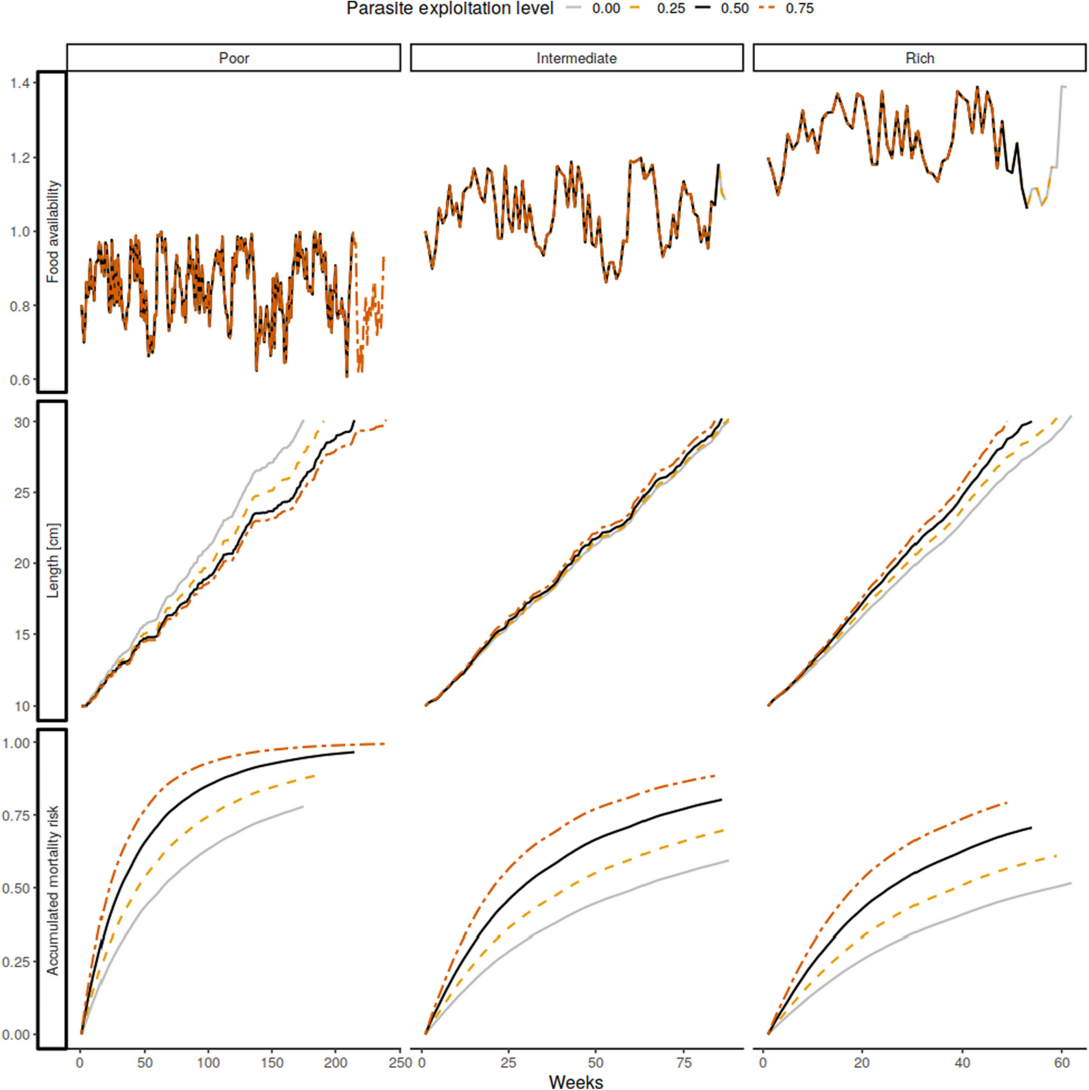
Under conditions of low food availability in the environment (top row), the optimal growth strategy for hosts experiencing high levels of parasite exploitation is to forage more intensely and therefore grow faster (middle row), while the opposite is true in rich environments; mortality is generally higher in the relatively poor environment due to higher foraging (risk‐taking) and increases with parasite exploitation level (bottom row).

In the group that experienced high food availability resembling laboratory conditions (right column of Figure 2), our model predicts faster growth with high‐cost parasites. The higher the parasite exploitation level, the faster the host growth and the higher the mortality risk. These patterns were also found under intermediate food availability (middle column of Figure 2) although the difference among exploitation levels was smaller. In the scenario with poor food availability (left column of Figure 2), the situation was reversed, with heavily parasitised hosts growing more slowly, while taking higher risks when foraging and thus having little chance of surviving.

### 3.3 Parasite fitness for different exploitation levels, in intermediate or final hosts

Parasite strategies are not optimised in our model, but we explore selection on exploitation levels for parasites, under two alternative scenarios assuming different life stages and transmission modes for the parasite.

**FIGURE 3:**
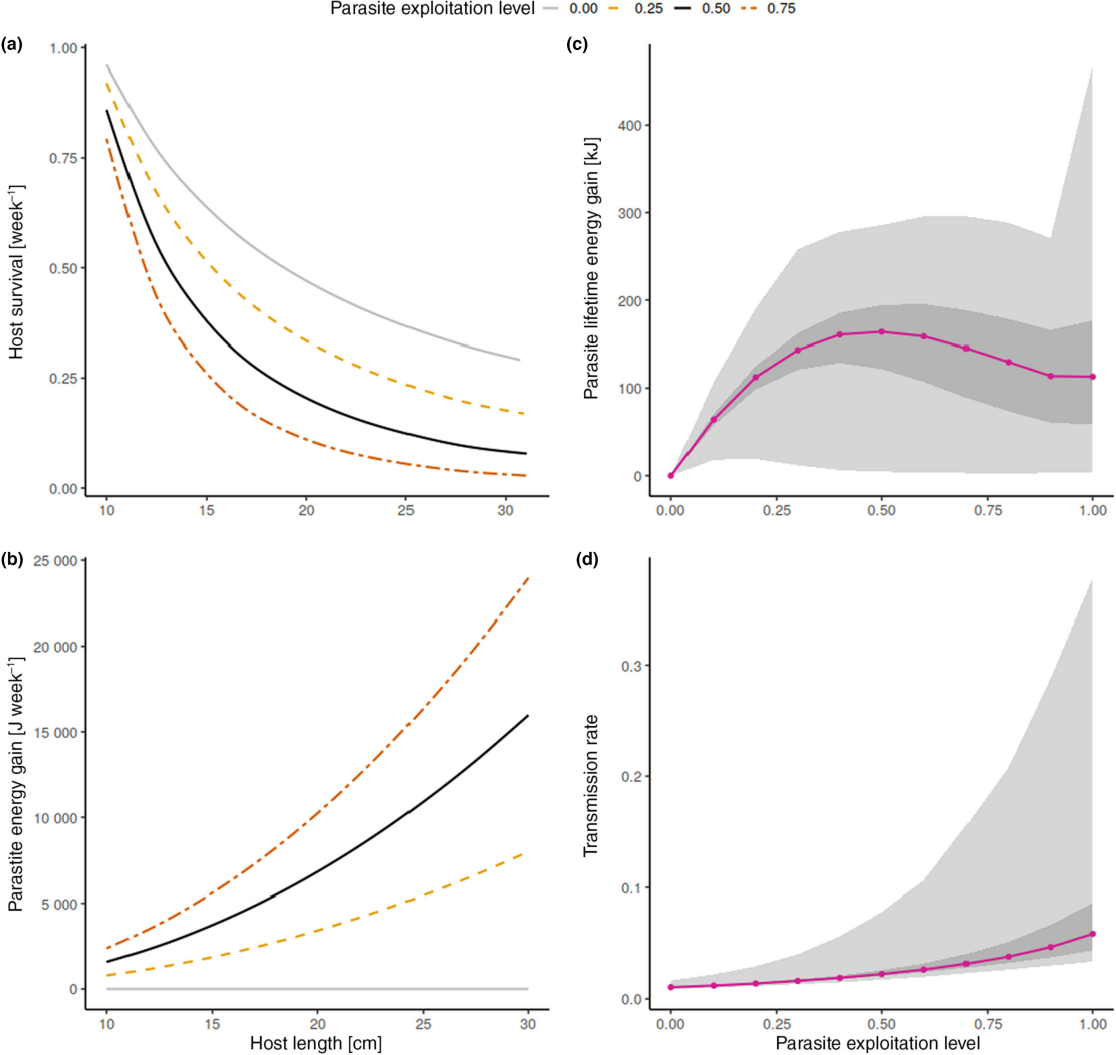
Effects of host responses on proxies of parasite fitness for different exploitation levels. (a) Mean host survival [week^−1^], with predation during foraging being the main cause of mortality in our model; (b) rate of energy gain for the parasite during host growth; (c) Parasite lifetime energy gain (parasite energy gain [J week^−1^]·host survival [week^−1^]), used here to approximate fitness for a parasite that needs its host to survive. (d) Expected transmission rate (−log(host survival [week^−1^])/host growth period [weeks]), used here to approximate fitness in those cases where the fish is an intermediate host and the parasite ready to be trophically transmitted to the next host. Violet circles represent median values, dark grey area represent the values from 0.25 to 0.75 quantile, while light grey areas represent the values from 0 to 1 quantile. Lines for (a) and (b) are smoothed using a generalised additive model for ease of reading.

A developing parasite would benefit from not killing its host until it is ready to leave it (in the case of an intermediate host) or have successfully reproduced (in the case of a final host). For such a parasite, lifetime energy gain [kJ] in the host can be used as a fitness proxy. According to our model, this proxy for fitness is maximised at an intermediate exploitation level (Figure 3c). In contrast, a trophically transmitted parasite that is ready to leave its intermediate host would benefit from increasing the probability that the host will be eaten by the next host in its life cycle. Here, a more suitable fitness proxy is transmission rate (here defined as −log(host survival [week^−1^])/host growth period [weeks]), and our model indicates that it increases with exploitation level (Figure 3d).

## 4 DISCUSSION

By modelling host responses to parasitism at the hormonal level, we find that the optimal response for juvenile parasitised hosts is to increase their feeding‐ and growth‐related hormone levels. The resulting higher foraging intensity, growth, metabolism and body condition come at the cost of increased predation risk. Furthermore, our model shows that gigantism or increased risk‐taking do not only reflect optimal responses in and for the host, but that several of these changes may also benefit the parasite.

Our results align with several former studies showing changes in metabolic rates and performance in infected hosts (Binning et al., 2013, 2017; Careau et al., 2012; McElroy & de Buron, 2014; Robar et al., 2011). Increased reserves coupled with growth enhancement may result in gigantism, where hosts increase in size following a parasitic infection. Gigantism has been reported in many taxa, for example *Daphnia* (Ebert et al., 2004), snails (Ballabeni, 1995) and fish (Arnott et al., 2000) and is often associated with host castration. According to the temporal storage hypothesis (Ebert et al., 2004) host castration benefits the parasite because it keeps the host growing, thereby accumulating reserves that can later be diverted into parasite reproduction. Even though gigantism is often associated with host castration, there are notable exceptions; three‐spined sticklebacks (*Gasterosteus aculeatus*) infected by the cestode *Schistocephalus solidus* display increased growth but no reduction in gonadal investment. They are also, like our model fish, heavier than uninfected fish, and show up to 17% increase in the weight of liver reserves (Arnott et al., 2000). One explanation may be that enhanced growth is a bet‐hedging strategy that helps hosts cope with the risk of starvation. The results from Arnott et al. (2000) need to be taken with some level of caution, however, as they were obtained in laboratory conditions where food was provided ad libitum, which corresponds to the ‘rich food availability’ environment in our model. Other studies of *G. aculeatus* infected with *S. solidus* under natural conditions, which are likely closer to the ‘poor food availability’ environment in our model, have shown a reduction in infected host growth and reproductive performance (Macnab et al., 2009). The fact that our model predicts increased growth of infected hosts when food availability is high, but the opposite patterns when food availability is poor, may help understand why gigantism is rarely observed in the wild (Barber et al., 2000; Fernandez & Esch, 1991; Taskinen, 1998).

The model described here optimises hormone levels from the perspective of the host only, and not the parasite. Our proxies for parasite fitness (lifetime energy gain or transmission rate), however, indicate that the host responses may also be adaptive for the parasite. The way in which selection favours parasite strategies that best balance extracting energy from the host while keeping it alive (also referred to as the ‘virulence‐transmission trade‐off’) has been well‐studied in the past decades (e.g. Alizon et al., 2009; Bull, 1994; Jensen et al., 2006; Mennerat et al., 2012). Our model also suggests that an intermediate exploitation level is best at solving this trade‐off, for parasites with a direct life cycle or for trophically transmitted parasites in pre‐infective stages (Figure 3c). For trophically transmitted parasites ready to reach their final host, fitness is maximised by exploiting the host as much as possible, inducing risky foraging behaviour, and hence increasing the chances of transmission to the next host (Figure 3d). The fact that host manipulation only occurs at the infective stage is well‐described elsewhere; repeatedly measuring hosts and comparing their responses at the pre‐ versus post‐infective stage is commonly used as a way to test whether altered host responses result from manipulation or are mere by‐products (e.g. Gabagambi et al., 2019; Hafer & Milinski, 2015; Poulin, 1994). The novelty here is that our model provides a mechanistic link for how switching from intermediate to high exploitation level as the parasite reaches infective stage may result in corresponding alterations in host behaviour, switching to higher foraging rates involving higher risk‐taking and resulting in higher predation rate.

Finally, not all behavioural or physiological changes following infection are explained by host compensatory mechanisms alone. Uncontroversial manipulation of hosts by parasites does exist; insects protecting the pupae of their parasitoids (Libersat et al., 2018 and references therein) or ‘zombie ants’ spreading spores of parasitic fungi (Hughes et al., 2011) are host manipulation, beyond doubt. Our results show nonetheless that simple physiological mechanisms should be considered as pre‐existing paths towards manipulation and that parasites would be selected for their ability to exploit compensatory responses in hosts whenever those benefit them (Lefèvre et al., 2008). Together with earlier studies we argue that the ‘energy drain hypothesis’ and the ‘parasite manipulation hypothesis’ need not be mutually exclusive and that some unresolved cases might be better understood by adopting a more holistic approach (e.g. Hafer & Milinski, 2016; Thomas et al., 2005). Behavioural changes following infection, even some of those that in some systems primarily benefit parasites, may in others be adaptive for infected hosts too.

## AUTHOR CONTRIBUTIONS

**Camilla Håkonsrud Jensen:** Conceptualization (equal); formal analysis (lead); investigation (lead); methodology (equal); writing – original draft (lead); writing – review and editing (equal). **Jacqueline Weidner:** Conceptualization (equal); formal analysis (equal); investigation (equal); methodology (equal); writing – original draft (equal); writing – review and editing (supporting). **Jarl Giske:** Conceptualization (equal); funding acquisition (lead); resources (lead); supervision (supporting); writing – original draft (equal). **Christian Jørgensen:** Conceptualization (equal); formal analysis (supporting); investigation (supporting); methodology (supporting); supervision (lead); writing – original draft (equal); writing – review and editing (supporting). **Sigrunn Eliassen:** Conceptualization (equal); formal analysis (supporting); investigation (supporting); project administration (lead); supervision (lead); writing – original draft (equal). **Adèle Mennerat:** Conceptualization (equal); investigation (supporting); supervision (supporting); writing – original draft (equal); writing – review and editing (lead).

### OPEN RESEARCH BADGES

This article has earned an Open Materials badge for making publicly available the components of the research methodology needed to reproduce the reported procedure and analysis. All materials are available at https://github.com/tinytyranid/HormoneModelParasite.

## Supporting information


Data S1

## Data Availability

The source code for the model used in the paper is freely available from GitHub: https://github.com/tinytyranid/HormoneModelParasite. The source code for an earlier version of the model is freely available from Zenodo: https://doi.org/10.5281/zenodo.4005943.
