# Supplementary material for: Adaptive host responses to infection can resemble parasitic manipulation
Source: Ecol Evol. 2023 Jul 15;13(7):e10318. doi: 10.1002/ece3.10318 (PMC10349281; doi:10.1002/ece3.10318)
Supplement: Supplementary file 1 — Data S1 [file ECE3-13-e10318-s001.docx]

**Supplementary Material**

**Extended Materials and Methods**

We use a stochastic dynamic optimisation model focusing on hormone regulation of growth, metabolism and foraging in juvenile fish. The model is an extension of Jensen et al. (2021) and Weidner et al. (2020). Identical to these works the complexity of the hormone system is simplified and represented by three different hormone functions: The Growth Hormone Function (GHF), the Thyroid Hormone Function (THF) and the Orexin Function (OXF). GHF regulates allocation to growth and OXF the fish’s appetite. The THF adjusts the standard metabolic rate (SMR) and the limit for maximum oxygen uptake. By adjusting these hormone function levels, the fish also affects its exposure to, and probability of escaping predators. The optimal hormone strategy balances the trade-off between growth and mortality in each model scenario. What follows is a written summary of the model. For more details on the implementation and the simplification process see Jensen et al. (2021) and Weidner et al. (2020).

Stochastic Dynamic Optimisation

We use stochastic dynamic optimisation, or state dependent programming (Mangel and Clark 1988, Clark and Mangel 2000), to find the optimal concentrations of the three hormone functions. To use optimisation, does not mean to assume that evolution by natural selection always will have reached the optimal solution. Rather, it allows us to find the solution that natural selection will be working towards – if selection only were to adapt to the much-simplified scenario of our model.

In this model, the fish have three states: (1) length, (2) reserves and (3) experienced food availability. We find the optimal combination of the three hormone functions (GHF, THF and OXF) that yields the highest fitness for the individual fish at the end of the growth period, given its current state combination. Technically this is done by an iteration from the last to the first time step in the model. In this approach, we are therefore only concerned with the fish’s current state and not how it got there. After the optimal combination of hormone function levels are found, for every time step and for every state combination, we simulate individual fish that are run through the same scenario from the first to the last time step (forward iteration) were they act in accordance with the optimal policy found in the backward iteration. (For more details, see *Optimisation* below and Weidner et al. 2020.)

Hormone Functions

Many hormone systems work by utilizing a negative feedback loop including a releasing hormone, a pituitary hormone, and an end hormone (Hiller-Sturmhöfel and Bartke 1998). The hypothalamus secretes a releasing hormone that follows the blood stream to the anterior pituitary. A pituitary hormone is then released and transported to the target gland, which secretes an end hormone. A constant negative feedback loop where the pituitary- and end hormones inhibit the secretion of the releasing hormone ensures a stable system. Here we simplify and combine hormones regulating different parts of the feedback mechanism into three “Hormone Functions”, affecting energy allocation to growth, metabolic rate, and foraging behaviour.

*GHF and Structural growth*

The Growth Hormone Function (GHF) affects energy allocation to growth ($\Delta W_{\text{structure}}$ [g week^-1^]):

$\Delta W_{\text{structure}}=(\frac{\gamma}{\gamma_{\text{max}}})\cdot k_{\text{growth}}\cdot W_{\text{structure}}$ **(S1)**

(See explanation of symbols in **Table S1**). Here, $\gamma$ [ng ml^-1^] is current GHF level, $\gamma_{\text{max}}$ [ng ml^-1^] is maximum possible GHF level, $k_{\text{growth}}$ [week^-1^] is the maximum limit for proportional increase in structural body mass in one time step [weeks], $W_{\text{structure}}$ [g] is structural weight calculated from Fulton’s condition factor for lean fish ($k_{\text{Fultons\_min}}$ [g cm^-1^]; Lambert & Dutil, 1997) and length ($L$ [cm]); $W_{\text{structure}}=k_{\text{Fultons\_min}}\cdot L^{3}$. Thus, a higher $\gamma$ leads to a higher growth per time step.

To find the energetic cost of growth ($C_{\text{growth}}$ [J]), both $\Delta W_{\text{structure}}$ and the energetic value of body structures, $d_{\text{structure}}$ [J g^-1^] (Holdway and Beamish 1984, Anthony et al. 2000, Fernandez et al. 2009), need to be taken into account: $C_{\text{growth}}=\Delta W_{\text{structure}}\cdot d_{\text{structure}}$.

*THF and Metabolism*

The Thyroid Hormone Function (THF) regulates the standard metabolic rate (SMR, $P_{\text{SMR}}$ [J min^-1^]):

$P_{\text{SMR}}=[1+(\frac{\tau}{\tau_{\text{max}}}-0.5)\cdot k_{\text{THF\_SMR}}]\cdot P_{\text{standard}}$ **(S2)**

Here, $\tau$ [ng ml^-1^] is current THF level, $\tau_{\text{max}}$ [ng ml^-1^] is the maximum THF level, $P_{\text{standard}}$ [J min^-1^] is the standard metabolic rate based on total weight ($W=W_{\text{structure}}+W_{\text{reserves}}$ [g]) at ${\tau_{\text{max}}}/2$ and $k_{\text{THF\_SMR}}$ [dimensionless] is the effect THF has on $P_{\text{standard}}$. Calculations of SMR are based on Clarke & Johnston (1999).

*THF and Oxygen use*

In addition to regulating SMR, THF also regulates maximum oxygen uptake ($A_{\text{max}}$ [J min^-1^]):

$A_{\text{max}}=[1+(\frac{\tau}{\tau_{\text{max}}}-0.5)\cdot k_{\text{THF\_scope}}]\cdot A_{\text{standard}}$ **(S3)**

Here, $A_{\text{standard}}$ [J min^-1^] is maximum O**_2_** uptake at ${\tau_{\text{max}}}/2$ and $k_{\text{THF\_scope}}$ [dimensionless] is the effect THF has on $A_{\text{standard}}$. During our simulations, $k_{\text{THF\_SMR}}$ is slightly higher than $k_{\text{THF\_scope}}$ (see **Table S1**). Calculations of maximum oxygen uptake are based on Claireaux et al. (2000).

The oxygen use ($P$ [J min^-1^]) is the sum of $P_{\text{SMR}}$, the energetic cost of digesting food (SDA, $P_{\text{SDA}}$ [J min^-1^]), the energetic cost of foraging ($P_{\text{foraging}}$ [J min^-1^], see **Eq. S7**) and conversion costs from intake to growth ($P_{\text{growth}}$ [J min^-1^]) and reserves ($P_{\text{reserves}}$ [J min^-1^]) (see Weidner et al. 2020 for details):

$P=P_{\text{SMR}}+P_{\text{foraging}}+P_{\text{SDA}}+P_{\text{reserves}}+P_{\text{growth}}$ **(S4)**

An increase in THF ($\tau$) results in higher SMR ($P_{\text{SMR}}$) with higher energetic costs and O_2_ use ($P$). On the other hand, THF also increases the maximum oxygen uptake ($A_{\text{max}}$) in the fish. In other words, THF both increases mortality through increased $P$, and decreases mortality due to an increase in the ratio between $P$ and $A_{\text{max}}$ (see **Eq. S15**).

*OXF and Foraging*

Appetite is controlled by the Orexin Function (OXF), which can be seen as a combination of the “hunger hormone” ghrelin (Dimaraki and Jaffe 2006), and the neuropeptide orexin. Target intake ($I$ [J min^-1^]) is proportional to the relative concentration of OXF ($\alpha/{\alpha_{\max}}$):

$I=\frac{\alpha}{\alpha_{\text{max}}}\cdot k_{\text{OXF}}\cdot P_{\text{structure}}$ **(S5)**

Here, $\alpha$ [pg ml^-1^] is the current OXF level, $\alpha_{\text{max}}$ [pg ml^-1^] is the maximum possible OXF level, $k_{\text{OXF}}$ [dimensionless] is the effect OXF has on intake and $P_{\text{structure}}$ [J min^-1^] is the SMR at ${\tau_{\text{max}}}/2$ based on structural weight of the fish.

The model environment is defined by the different food availabilities for the fish ($E$ [dimensionless]). There will always be some food, but the fish must spend more time foraging to reach the same target intake ($I$ [J min^-1^]), when the food availability is poor:

$B_{\text{foraging}}=\frac{I}{P_{\text{stucture}}\cdot E}$ **(S6)**

Here, $B_{\text{foraging}}$ [dimensionless, given in multiples of $P_{\text{structure}}$] is the foraging activity required to reach $I$. A higher OXF level ($\alpha$) thus leads to a hungrier fish and a higher energy intake, but at the cost of higher exposure to predators depending on current food availability (see **Eq. S14**).

The energetic cost of foraging ($P_{\text{foraging}}$ [J min^-1^]) is found by taking into account $B_{\text{foraging}}$ as well as SMR based on total weight ($P_{\text{standard}}$) and a scaling constant for foraging ($k_{\text{foraging}}$ [dimensionless]):

$P_{\text{foraging}}=k_{\text{foraging}}\cdot B_{\text{foraging}}\cdot P_{\text{standard}}$ **(S7)**

Thus, since foraging takes longer when the food availability is low (low $E$), it carries a higher energy cost, than foraging when the food availability is high (high $E$).

Parasite exploitation of host

The only characteristic of the model parasite is that it takes energy from the host. For simplicity, the exploitation level is kept stable throughout the simulation. Since there is no parasite strategy in the model, but the changes in host mortality, physiology or behaviour that follow represent host responses to increased energetic demands due to infection. We make no assumptions about the life history of the parasite, or whether it is a micro- or macroparasite. Inter- and intraspecific competition between parasites is also not considered and we make no assumptions regarding the number of parasites infecting the host (for ease of reading we will use singular form in this supplement).

The energetic cost of being parasitised [J min^-1^] is:

$P_{\text{parasite}}=P_{\text{structure}}\cdot k_{\text{parasite}}$ **(S8)**

where $k_{\text{parasite}}$ [dimensionless] is the exploitation level of the parasite and $P_{\text{structure}}$ [J min^-1^] is the standard metabolic rate of the fish based on structural weight at an intermediate level of THF (*τ*_max_/2 [ng ml^−1^] where $\tau_{\text{max}}$ is the maximum THF level [ng ml^−1^]). The energetic cost of being parasitised thus increases with the structural weight of the fish, while the exploitation level of the parasite is kept constant throughout the simulation.

Host defence against parasites

The model fish has no means of getting rid of the parasite, and its only option is to optimise the physiological response to the new situation (i.e., tolerance). This involves changing the hormonal strategy that regulates behaviour and energetics to maximise its survival probability through the juvenile phase.

Reserves

Together with length ($L$) and experienced food availability ($E$*),* reserves ($R$) is one of the three states in the model. Fish may cover the energetic cost of being parasitised by increasing food intake ($I$ [J min-1]) or draining energy reserves ($R$ [J]). The host's reserves at the next time step ($t+1$) depend on foraging behaviour and energy allocation in the current time step:

$R\left( t+1 \right)=R\left( t \right)-C_{\text{growth}}+\left( I- P_{\text{SDA}}-P_{\text{SMR}}-P_{\text{foraging}}-P_{\text{parasite}}-P_{\text{growth}}-P_{\text{reserves}} \right) \cdot t_{\text{duration}}$ **(S9)**

Here $R(t)$ and $R(t+1)$ are the reserves $R$ [J] at the beginning and end of the time step $t$. Bioenergetic rates must be multiplied by the duration of a time step, $t_{\text{duration}}$ [min]. The expression $(I-P_{\text{SDA}}-P_{\text{SMR}}-P_{\text{foraging}})$ can be viewed as the energetic surplus available for growth [J week^-1^], or the energy left from intake after metabolism, digestion and activity are accounted for.

Starvation

In the current version of the model host survival $S$ [week^-1^] depends not only on total mortality $M$ [year^-1^] and reserves $R$ but also a predefined starvation level *k*_starvation_ [proportion of energy reserves]. In fish with adequate reserves weekly survival probability is $e^{{-M}/{52}}$, but if $R$ drops below $k_{\text{starvation}}\cdot R_{\text{max}}$ fish survival rapidly declines with relative energy reserves $R/{R_{\text{max}}}$:

$S= e^{{-M}/{52}}\cdot(\frac{1}{k_{\text{starvation}}})\cdot(\frac{R}{R_{\text{max}}})$ **(S10)**

Food availability

The food availability ($E$ [dimensionless]) varies gradually over time (for more details see Jensen et al. 2021). Consecutive values of food availability are autocorrelated. Fish cannot migrate; only respond to food availability changes by adjusting their hormone profile. Even if food availability is poor, fish can always find some food, but must spend more time and energy to do so, at the cost of increased predator exposure (see **Eq. S6** & **S14**).

Food availability follows a normal distribution and intermediate food availability therefore occurs more frequently than poor and rich. To find $E$ in week $t$ ($E(t)$), we use an autocorrelated process modified from Ripa and Lundberg (1996):

$E\left( t \right)=k_{\text{E\_sd}}\cdot\left[ E\left( t-1 \right)\cdot k_{\text{E\_autocorr}}+\text{normal}\left( 0,1 \right)\cdot\sqrt{1-k_{\text{E\_autocorr}}^{2}} \right]+1, E\in[\text{E}_{\text{min}},\text{E}_{\text{max}}]$ **(S11)**

Here $E(t-1)$ is the relative food availability (where the average is 1) in the previous time step $t-1$. $\text{normal}(0,1)$ is a random number drawn from a normal distribution with mean of 0 and a standard deviation of 1. $\text{k}_{\text{E\_autocorr}}$ is the autocorrelation constant: For $k_{\text{E\_autocorr}}=1$ food availability is constant, while $k_{\text{E\_autocorr}}=0$ results in a current food availability that does not depend on the previous food level. We consider a scenario where $0<k_{\text{E\_autocorr}}<1$ and the food availability is positively autocorrelated between time steps. $k_{\text{E\_sd}}$ is the number of standard deviations that correspond to the richest and poorest food availability in the simulation. When implemented, the distribution is capped between $E_{\text{min}}$ and $E_{\text{max}}$, representing the poorest and richest food availability respectively.

Mortality

The total instantaneous mortality rate ($M$ [year^-1^]) is divided into five main components that all are affected differently by hormone function levels and fish body length: (1) size-independent mortality ($m_{\text{fixed}}$ [year^-1^]), (2) size-dependent mortality ($M_{\text{size}}$ [year^-1^]), (3) foraging-related mortality ($M_{\text{foraging}}$ [year^-1^]), (4) scope-related mortality ($M_{\text{scope}}$ [year^-1^]), as well as an (5) active-while-vulnerable mortality component ($M_{\text{foraging}\times\text{scope}}$ [year^-1^]):

$M=m_{\text{fixed}}+M_{\text{size}}+M_{\text{foraging}}+M_{\text{scope}}+M_{\text{foraging}\times\text{scope}}$ **(S12)**

The probability that the fish will survive the current time step is $S=\exp({-M}/{52})$ [week^-1^].

The size-independent mortality $m_{\text{fixed}}$ is unaffected by fish length or hormone function levels, and is kept at a stable, low level. This low level is chosen as we assume that most of the mortality affecting a small fish is highly dependent on size.

Size-dependent mortality $M_{\text{size}}$ decreases with increasing fish length ($L$) according to

$M_{\text{size}}=m_{\text{size}}\cdot L^{x_{\text{size}}}$ **(S13)**

where $m_{\text{size}}$ [year^-1^] and $x_{\text{size}}$ [dimensionless] are the size-dependent mortality coefficient and exponent, respectively. The size-dependent mortality interacts with all other mortality components in the model, except the baseline mortality (see **Eq. S12**). Thus, a small fish is more susceptible to predation than a bigger fish when keeping everything else equal.

Foraging mortality $M_{\text{foraging}}$ is connected to the foraging activity of the fish ($B_{\text{foraging}}$), which is affected by the food availability of the environment the fish is currently in as well as the OXF level of the fish (see **Eq. S5** & **S6**). For example, if we have two individuals with the same OXF levels and one experiencing low and the other high food availability, then the individual with poor food availability will also experience higher foraging mortality. This is because it will need to spend more time and energy foraging (a higher $B_{\text{foraging}}$) to satiate the same hunger level ($I$).

$M_{\text{foraging}}=m_{\text{foraging}}\cdot M_{\text{size}}\cdot B_{\text{foraging}}^{x_{\text{foraging}}}$ **(S14)**

where $m_{\text{foraging}}$ [year^-1^] and $x_{\text{foraging}}$ [dimensionless] are the foraging mortality coefficient and exponent respectively.

The scope-related mortality $M_{\text{scope}}$ is affected by the ratio between the used oxygen ($P$) and the maximum oxygen uptake set by THF ($A_{\text{max}}$). It is important to note that THF does not only increase aerobic scope, but also the actual O_2_ use through the positive effect of THF on SMR ($P_{\text{SMR}}$; see **Eq. S2**-S**4**). The higher $P$ is in relation to $A_{\text{max}}$, the lower is the individual’s probability to escape from a predator, and thus the ratio increases the fish’s scope-related mortality. In other words, a high $M_{\text{scope}}$ means that the fish has a lower potential for escaping a predator.

$M_{\text{scope}}=m_{\text{scope}}\cdot M_{\text{size}}\cdot{\frac{P}{A_{\text{max}}}}^{x_{\text{scope}}}$ **(S15)**

where $m_{\text{scope}}$ [year^-1^] and $x_{\text{scope}}$ [dimensionless] are the scope mortality coefficient and exponent respectively.

The active-while-vulnerable mortality component $M_{\text{foraging}\times\text{scope}}$ represents the interaction between foraging and scope mortality. It can be viewed as the fish’s potential to escape a predator while foraging, where a higher interaction mortality equates to a poorer potential for escape. The potential to escape depends on both the time and energy spent while foraging.

$M_{\text{foraging}\times\text{scope}}=\frac{m_{\text{foraging}\times\text{scope}}\cdot M_{\text{foraging}}\cdot M_{\text{scope}}}{M_{\text{size}}}$ **(S16)**

where $m_{\text{foraging}\times\text{scope}}$ [year^-1^] is the active-while-vulnerable coefficient.

Optimisation

In this model scenario, the model fish needs to grow from 10 to 30 cm, and this is achieved by the proximate mechanism of hormone function regulation. The fish that die or are not able to reach 30 cm are given a terminal fitness score of 0, while fish that do grow up are given a score of 1. In other words, the fish are only “rewarded” if they reach 30 cm within the time limit imposed by the model.

To find the optimal hormone function strategy we did a backward iteration from the final time step in the model, and the optimal hormone function concentrations were calculated for each week according to the fishes’ three states: (1) length, (2) reserve fullness and (3) experienced food availability. After the optimal hormone strategy for every week was calculated, the fish was then run through a forward iteration starting in the first week where the model fish behave according to the decision matrix (Mangel 2003) calculated in the backwards iteration of the model.

The optimisation algorithm used in the backward iteration of the model finds the optimal combination of GHF ($\gamma$), THF ($\tau$) and OXF ($\alpha$) that yields the highest possible expected fitness at the end of the growth period ($F(t,L,R,E)$) according to the three states:

$F(t,L,R,E)=\max_{\gamma,\tau,\alpha}\{S(L,R,E,\gamma,\tau,\alpha)\cdot\sum_{E'} \langle\text{prob}(E'\mid E)\cdot F[t+1,L'(\gamma,\tau,\alpha),R'(\gamma,\tau,\alpha),E']\rangle\}$ **(S17)**

Here $F(t,L,R,E)$ is the expected fitness at the end of the growth period of an individual fish at time $t$ of length $L$, reserves $R$ and that experience food availability $E$. Further, $S(L,R,E,\gamma,\tau,\alpha)$ is the survival probability (see *Mortality*) of an individual with states $L$, $R$ and $E$*,* and with hormone function levels $\gamma$*,* $\tau$ and $\alpha$. The autocorrelation parameter $\text{prob}(E'\mid E)$ is the probability that the next food availability is $E'$ given that the current food availability is $E$*.* Similarly, $F[t+1,L'(\gamma,\tau,\alpha),R'(\gamma,\tau,\alpha),E']$ is the expected fitness at the end of the growth period of an individual fish at time $t+1$ with states $L'$, $R'$ and $E'$and hormone strategy with hormone function levels $\gamma$*,* $\tau$ and $\alpha$. Thus, for every combination of $L$, $R$ and $E$ this procedure will find the corresponding optimal combination of $\gamma$*,* $\tau$ and $\alpha$.

**Table S1:** Model parameters, variables and functions referred to in the text. New or changed parameters since Jensen et al. (2021) is marked with light grey.

|  | **Value** | **Unit** | **Definition** |
| --- | --- | --- | --- |
| **PARAMETERS** | | | |
| $d_{\text{structure}}$ | 4 000 | J g^-1^ | Energetic value of body structures |
| $E_{\text{max}}$ | 1.40 |  | Maximum food availability |
| $E_{\text{min}}$ | 0.36 |  | Minimum food availability |
| $k_{\text{E\_autocorr}}$ | 0.80 |  | Autocorrelation constant for the food availability |
| $k_{\text{E\_sd}}$ | 0.35 |  | The number of standard deviations that corresponds to $E_{\text{min}}$ and $E_{\text{max}}$ |
| $k_{\text{foraging}}$ | 0.2 |  | Scaling constant for energetic cost of foraging |
| $k_{\text{Fultons\_min}}$ | 0.85 * 10^-8^ | g cm^-3^ | Fulton’s condition factor for lean fish |
| $k_{\text{growth}}$ | 0.28 | week^-1^ | Maximum possible proportion of growth in one time step |
| $k_{\text{OXF}}$ | 8.5 |  | The effect OXF has on intake |
| $k_{\text{parasite}}$ | 0.0 – 1.0 |  | The parasite exploitation level |
| $k_{\text{starvation}}$ | 0.01 |  | The starvation level of the fish. When reserves fall below this proportion, the survival goes towards 0. |
| $k_{\text{THF\_scope}}$ | 0.2 |  | Effect of THF on $A_{\text{standard}}$ |
| $k_{\text{THF\_SMR}}$ | 0.25 |  | Effect of THF on $P_{\text{standard}}$ |
| $m_{\text{fixed}}$ | 0.01 | year^-1^ | Size-independent mortality |
| $m_{\text{foraging}}$ | 0.03 | year^-1^ | Foraging mortality coefficient |
| $m_{\text{foraging}\times\text{scope}}$ | 1.2 | year^-1^ | Active-while-vulnerable mortality coefficient |
| $m_{\text{scope}}$ | 1.3 | year^-1^ | Scope mortality coefficient |
| $m_{\text{size}}$ | 1.3 | year^-1^ | Size-dependent mortality coefficient |
| $x_{\text{foraging}}$ | 3 |  | Foraging mortality exponent |
| $x_{\text{scope}}$ | 2.7 |  | Scope mortality exponent |
| $x_{\text{size}}$ | -0.75 |  | Size-dependent mortality exponent |
| $\alpha_{\text{max}}$ | 2 500 | pg ml^-1^ | Maximum OXF level |
| $\gamma_{\text{max}}$ | 200 | ng ml^-1^ | Maximum GHF level |
| $\tau_{\text{max}}$ | 5 | ng ml^-1^ | Maximum THF level |
| **VARIABLES** | | | |
| $A_{\text{max}}$ |  | J min^-1^ | Maximum possible oxygen uptake under the influence of THF |
| $A_{\text{standard}}$ |  | J min^-1^ | Maximum possible oxygen uptake at ${\tau_{\text{max}}}/2$ |
| $B_{\text{foraging}}$ |  | given in multiples of $P_{\text{structure}}$ | Foraging activity required to reach $I$ |
| $C_{\text{growth}}$ |  | J | Energetic cost of growth |
| $E$ |  |  | Food availability |
| $F$ |  |  | Fitness |
| $I$ |  | J min^-1^ | Target intake |
| $L$ |  | cm | Body length |
| $M_{\text{foraging}}$ |  | year^-1^ | Foraging mortality |
| $M_{\text{foraging}\times\text{scope}}$ |  | year^-1^ | Active-while-vulnerable mortality |
| $M_{\text{scope}}$ |  | year^-1^ | Scope mortality |
| $M_{\text{size}}$ |  | year^-1^ | Size-dependent mortality |
| $P$ |  | J min^-1^ | Oxygen use |
| $P_{\text{foraging}}$ |  | J min^-1^ | The energetic cost of foraging |
| $P_{\text{growth}}$ |  | J min^-1^ | Conversion costs from intake to growth |
| $P_{\text{parasite}}$ |  | J min^-1^ | The energetic cost of being parasitised |
| $P_{\text{reserves}}$ |  | J min^-1^ | Conversion costs from reserves to growth |
| $P_{\text{SDA}}$ |  | J min^-1^ | The energetic cost of digesting food |
| $P_{\text{SMR}}$ |  | J min^-1^ | SMR under the influence of THF |
| $P_{\text{standard}}$ |  | J min^-1^ | SMR at ${\tau_{\text{max}}}/2$ based on total weight ($W$) |
| $P_{\text{structure}}$ |  | J min^-1^ | SMR at ${\tau_{\text{max}}}/2$ based on structural weight of the fish ($W_{\text{structure}}$) |
| $R$ |  | J | Reserves |
| $S$ |  | year^-1^ | Survival probability |
| $t$ |  | week | Current time step |
| $W$ |  | g | Total weight |
| $W_{\text{structure}}$ |  | g | Structural weight |
| $\Delta W_{\text{structure}}$ |  | g week^-1^ | Growth |
| $W_{\text{reserves}}$ |  | g | Weight of reserves |
| $\alpha$ |  | pg ml^-1^ | OXF level |
| $\gamma$ |  | ng ml^-1^ | GHF level |
| $\tau$ |  | ng ml^-1^ | THF level |
| **FUNCTIONS** | | | |
| $\text{normal}(0,1)$ |  |  | Random number drawn from a normal distribution with mean of 0 and a standard deviation of 1 |
| $\text{prob}(E'\mid E)$ |  |  | The probability of the next environment being $E'$ given that the current environment is $E$ |

**Note:** Some parameters are from the literature and references to these papers can be found in the supplement when they are used in equations. The remaining parameters was found through a parametrisation process by tuning the unknown parameters so that the results show yearly mortality values within the normal range, as well as dynamics in hormone function levels.

**References**

Anthony, J. A. et al. 2000. Lipid content and energy density of forage fishes from the northern Gulf of Alaska. - J. Exp. Mar. Biol. Ecol. 248: 53–78.

Claireaux, G. et al. 2000. Influence of water temperature and oxygenation on the aerobic metabolic scope of Atlantic cod (*Gadus morhua*). - J. Sea Res. 44: 257–265.

Clark, C. W. and Mangel, M. 2000. Dynamic State Variable Models in Ecology: Methods and Applications. - Oxford University Press.

Clarke, A. and Johnston, N. M. 1999. Scaling of metabolic rate with body mass and temperature in teleost fish. - J. Anim. Ecol. 68: 893–905.

Dimaraki, E. V. and Jaffe, C. A. 2006. Role of endogenous ghrelin in growth hormone secretion, appetite regulation and metabolism. - Rev. Endocr. Metab. Disord. 7: 237–249.

Fernandez, D. A. et al. 2009. Energy density of sub-Antarctic fishes from the Beagle Channel. - Fish Physiol. Biochem. 35: 181–188.

Hiller-Sturmhöfel, S. and Bartke, A. 1998. The endocrine system: An overview. - Alcohol Health Res. World 22: 153–164.

Holdway, D. A. and Beamish, F. W. H. 1984. Specific growth rate and proximate body composition of Atlantic cod (*Gadus morhua* L.). - J. Exp. Mar. Biol. Ecol. 81: 147–170.

Jensen, C. H. et al. 2021. Hormonal adjustments to future expectations impact growth and survival in juvenile fish. - Oikos 130: 41–51.

Lambert, Y. and Dutil, J. D. 1997. Condition and energy reserves of Atlantic cod (*Gadus morhua*) during the collapse of the northern Gulf of St. Lawrence stock. - Can. J. Fish. Aquat. Sci. 54: 2388–2400.

Mangel, M. 2003. Environment and longevity: The demography of the growth rate. - Popul. Dev. Rev. 29: 57–70.

Mangel, M. and Clark, C. W. 1988. Dynamic Modeling in Behavioral Ecology. - Princeton University Press.

Ripa, J. and Lundberg, P. 1996. Noise colour and the risk of population extinctions. - Proc. Biol. Sci. 263: 1751–1753.

Weidner, J. et al. 2020. Hormones as adaptive control systems in juvenile fish. - Biol. Open 9: bio046144.
